# Novel gene-specific Bayesian Gaussian mixture model to predict the missense variants pathogenicity of Sanfilippo syndrome

**DOI:** 10.1038/s41598-024-62352-0

**Published:** 2024-05-27

**Authors:** Eman E. A. Mohammed, Alaaeldin G. Fayez, Nabil M. Abdelfattah, Ekram Fateen

**Affiliations:** 1 Medical Molecular Genetics Department, Human Genetics and Genome Research Institute, National Research Centre, Giza, Egypt; 2 Molecular Genetics and Enzymology Department, Human Genetics and Genome Research Institute, National Research Centre, Giza, Egypt; 3 Nasser’s Institute for Research and Treatment, Cairo, Egypt; 4 Biochemical Genetics Department, Human Genetics and Genome Research Institute, National Research Centre, Giza, Egypt

**Keywords:** Machine-learning model, Sanfilippo syndrome, Missense variants, Pathogenicity prediction, Computational biology and bioinformatics, Genetics

## Abstract

MPS III is an autosomal recessive lysosomal storage disease caused mainly by missense variants in the *NAGLU, GNS, HGSNAT*, and *SGSH* genes. The pathogenicity interpretation of missense variants is still challenging. We aimed to develop unsupervised clustering-based pathogenicity predictor scores using extracted features from eight in silico predictors to predict the impact of novel missense variants of Sanfilippo syndrome. The model was trained on a dataset consisting of 415 uncertain significant (VUS) missense *NAGLU* variants. Performance The SanfilippoPred tool was evaluated by validation and test datasets consisting of 197-labelled *NAGLU* missense variants, and its performance was compared versus individual pathogenicity predictors using receiver operating characteristic (ROC) analysis. Moreover, we tested the SanfilippoPred tool using extra-labelled 427 missense variants to assess its specificity and sensitivity threshold. Application of the trained machine learning (ML) model on the test dataset of labelled *NAGLU* missense variants showed that SanfilippoPred has an accuracy of 0.93 (0.86–0.97 at CI 95%), sensitivity of 0.93, and specificity of 0.92. The comparative performance of the SanfilippoPred showed better performance (AUC = 0.908) than the individual predictors SIFT (AUC = 0.756), Polyphen-2 (AUC = 0.788), CADD (AUC = 0.568), REVEL (AUC = 0.548), MetaLR (AUC = 0.751), and AlphMissense (AUC = 0.885). Using high-confidence labelled *NAGLU* variants, showed that SanfilippoPred has an 85.7% sensitivity threshold. The poor correlation between the Sanfilippo syndrome phenotype and genotype represents a demand for a new tool to classify its missense variants. This study provides a significant tool for preventing the misinterpretation of missense variants of the Sanfilippo syndrome-relevant genes. Finally, it seems that ML-based pathogenicity predictors and Sanfilippo syndrome-specific prediction tools could be feasible and efficient pathogenicity predictors in the future.

## Introduction

Sanfilippo syndrome is named after Dr. Sylvester Sanfilippo, who discovered the cause of this disease in 1963. This disorder is due to the absence of specific lysosomal enzymes essential for breaking down glycosaminoglycans (GAGs), called heparan sulfate. Sanfilippo syndrome, also known as Mucopolysaccharidoses III (MPS III), is an autosomal recessive metabolic disorder caused by variants in genes responsible for the degradation of GAGs, heparan sulfate (HS), located in the extracellular membrane. The lysosomal accumulation of undegraded heparan sulfate leads to cellular dysfunction and pathology in several organs, with severe central nervous system degeneration resulting in dementia and behavioral abnormalities as the main clinical characteristics of MPS III^1^. MPS III has four different subtypes designed as MPS III type A (MPS IIIA), MPS III type B (MPS IIIB), MPS III type C (MPS IIIC), and MPS III type D (MPS IIID), caused by the deficiency of one of the four lysosomal specific enzymes, heparan N-sulfatase (sulfamidase) (SGSH), alpha-N-acetylglucosaminidase (NAGLU), acetyl CoA-alpha-glucosaminidase N-acetyltransferase (HGSNAT) and N-acetylglucosamine-6-sulfatase (GNS), respectively. All subtypes of MPS III have similar clinical phenotypes and are common in the elevation of urinary heparan sulphate level^2^.

MPS IIIB is caused by over 170 variants in the N-acetyl- alpha-D-glucosaminidase (*NAGLU*) gene, an 8.5 kb, consists of 6 exons, and is localised on chromosome 17q21. The *NAGLU* gene encodes the α-N-acetylglucosaminidase enzyme of 743 amino acids, with a 20–23-aa signal peptide immediately preceding the amino terminus of the enzyme and with six potential N-glycosylation sites, which catalyses the removal of the N-acetylglucosamine residues from the non-reducing terminal of heparan sulphate by lysosomal degradation^3^. Pathogenic *NAGLU* variants reduce the activity of this enzyme and result in lysosomal accumulation of both non-degraded and partially degraded heparan sulfate^4^. A crystalline *NAGLU* enzyme is composed of three domains (I, II, and III). Amino acids 24–126 form a small α/β domain in Domain I. Domain II contains the catalytic residues, amino acids 127–467 form an (α/β)8 domain. Amino acids 468–743 form an α-helical domain in Domain III, the active site is located between Domains II and III^5^. MPS IIIB patients are diagnosed by quantitative measurement of GAGs and two-dimensional electrophoresis of urinary GAGs^6^. The enzyme activity, α-N-acetylglucosaminidase (NAGLU) (MPS IIIB; EC 3.2.1.50) was assayed in the plasma using fluorogenic substrates^7^. The normal range of NAGLU activity is 10–45 μmol/l/h and for the normal cases of MPS III, the electrophoretic separation of urinary GAGs should not show heparan (H) or heparan sulfate (HS) spots.

Interpreting the effect of missense VUS variants is challenging, especially since functional study of the pathogenicity of large numbers of variants is costly and time-consuming. Consequently, many algorithms were developed to accurately predict the deleteriousness of VUS variants and improve clinical diagnosis, patient management, and personalised treatment. Peterson et al.^8^ pointed out that computational tools for variant impact prediction rely on data-derived expert knowledge or data-driven machine-learning algorithms^8^.

While the current computational tools provide inconsistent variant interpretation with each other^9,10^, these tools neglect the gene function nature and distinct disease pathogenicity modes, disease-specific tools are in high demand now. It is well known that the complementary approach of individual computational tools has the best variant interpretation power deriving from multiple variant features and prediction algorithms.

Currently, genome-wide prediction tools have low specificity; Zhang et al.^11^ pointed out that classifying variants as pathogenic or not, without reference to a specific disease or mechanism, may not perform as well as those that separate gene-disease relations^11^. Moreover, Ruklisa et al.^12^ showed that considering gene-disease relevance in a classification computational model improves variant interpretation^12^. In addition, Richards et al.^13^ noted that genome-wide prediction tools gave over a handful for limiting false-positive assertions (type I error) and providing over-prediction of disease-causing variants^13^.

This study aims to construct a model capable of classifying pathogenic and benign *NAGLU* missense variants using unsupervised clustering. To overcome the drawbacks of genome-wide prediction tools, we combined several computational scores that consider gene-disease relations by taking missense variants, which are common in MPS III. Moreover, we tested this model on the four subtypes of MPS III relevantgenes. Finally, we constructed the Excel-based “SanfilippoPred” tool that combines the four genes associated with MPS III. “SanfilippoPred” demonstrated better accuracy than the individual genome-wide tools to distinguish between the pathogenic variants and the benign variants in MPS III-relevant genes.

## Results

Data on 612 variants of the *NAGLU* gene were included in the analysis. Out of 612 variants, 197 variants were labelled, of which 96 variants (48.7%) were likely pathogenic or pathogenic and 101 variants were benign or likely benign (51.3%). Genome-wide variant pathogenicity predictors (features) included Grantham’s distance, Sneath’s score, SIFT, PolyPhen-2, CADD, REVEL, MetaLR, and the MutationAssessor. The statistical summary of the 612 variant scores presented in Table 1.

**Table 1:** Descriptive statistics for the 612 variants according to their calculated features.

	Features*							
	Grantham	Sneath	SIFT	PolyPhen-2	CADD	REVEL	MetaLR	MutationAssessor
Min	5.000	5.000	0.000	0.000	0.000	0.124	0.000	0.000
Max	215.000	43.000	1.000	1.000	32.000	0.995	0.997	0.951
Range	210.000	38.000	1.000	1.000	32.000	0.871	0.997	0.951
Median	64.000	23.000	0.040	0.595	23.000	0.576	0.915	0.671
Mean	76.761	23.279	0.188	0.526	19.466	0.590	0.882	0.610
SD	44.707	9.186	0.269	0.420	8.077	0.263	0.112	0.291

*SIFT, sorting intolerant fron tolerant; CADD, combined annotation dependent depletion, PolyPhen, polymorphism phenotyping; REVEL, rare exome variant ensembl learner.

**Figure 1:**
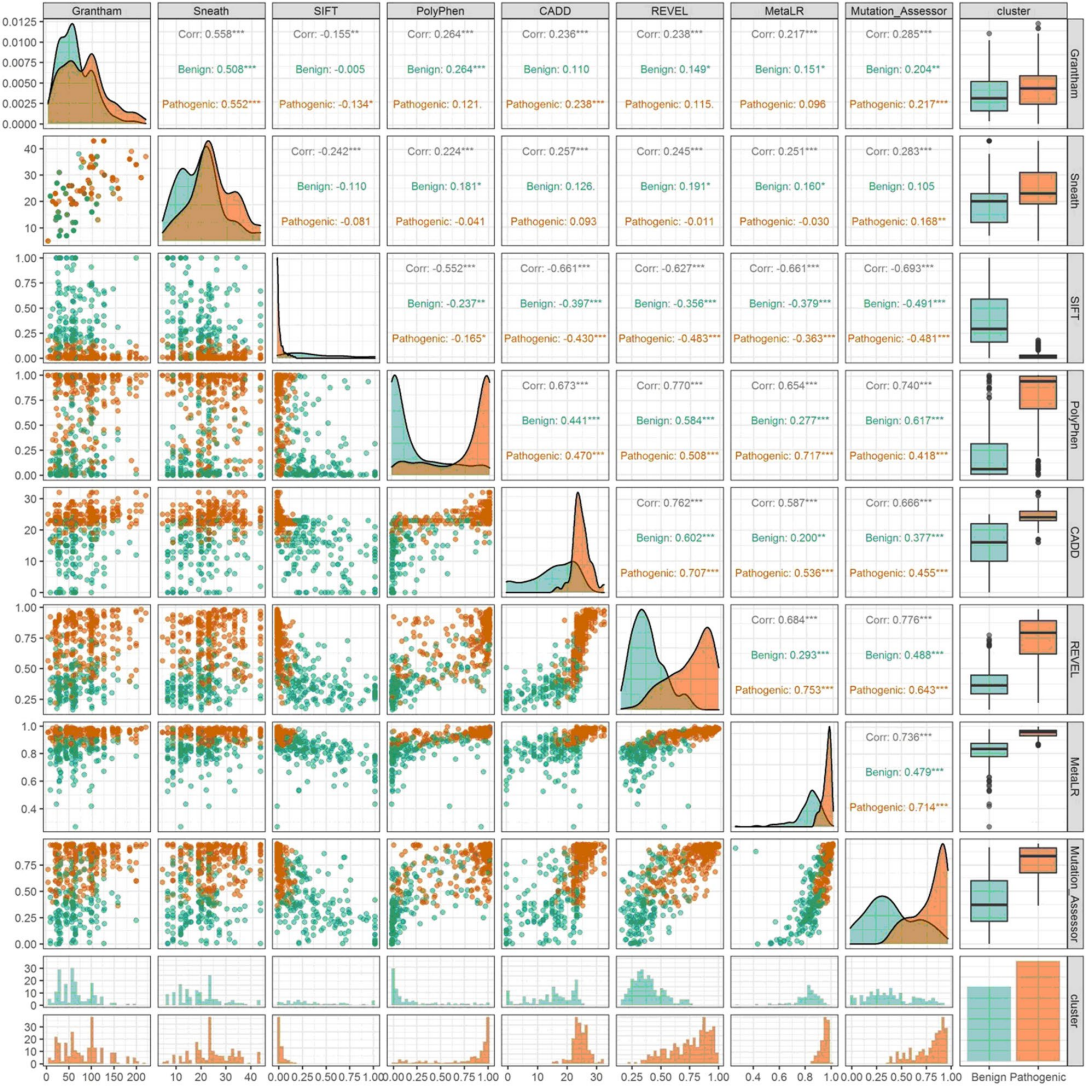
Correlation and scatterplot matrix between scores for each cluster.

The posterior means, 50th percentiles, and 95% credible intervals for each variable in cluster centroids of the full model (including all the variables) and the parsimonious model for 415 VUS (unlabeled) NAGLU missense variants are presented in Table 2. For the full model, 238 out of 415 VUS variants (57.3%) were classified as pathogenic, and 177 out of 415 (42.7%) were classified as benign variants. It is noted that the sampler converged to values consistent with each score value denoting a benign or pathogenic variant. The full model had a validation accuracy of 89%, a sensitivity of 88%, a specificity of 89%, a positive predictive value (PPV) of 90%, and a negative predictive value (NPV) of 88%. The AUC was 0.93 (95% CI 0.87–0.98). On the test set, the full model achieved an accuracy of 93%, a specificity of 93%, a sensitivity of 92%, a PPV of 91%, and an NPV of 94% (Table 3). The clustering of unlabeled data using the full model presented in Fig. 1.

**Table 2:** Cluster centroid estimates obtained by BGMM using data of the 415 VUS variants.

	Benign				Pathogenic			
	Mean (± SD)	2.50%	50.0%	97.5%	Mean (± SD)	2.50%	50.0%	97.5%
Full model								
Cluster weights (ω_k_):	0.43 (± 0.03)	0.39	0.43	0.49	0.57 (± 0.03)	0.51	0.58	0.61
Cluster means (µ_k_):								
Grantham’s distance	63.4 (± 3.09)	57.8	63.1	68.7	82.4 (± 3.61)	76.3	82.4	88.3
Sneath index	19.7 (± 0.76)	18.4	19.7	20.9	24.6 (± 0.70)	23.5	24.6	25.7
SIFT score	0.38 (± 0.04)	0.33	0.38	0.43	0.03 (± 0.03)	0.02	0.03	0.03
Polyphen-2	0.22 (± 0.05)	0.17	0.21	0.26	0.77 (± 0.05)	0.73	0.78	0.82
CADD	15.0 (± 0.96)	13.7	14.9	16.1	24.4 (± 0.76)	24.0	24.4	24.8
REVEL score	0.39 (± 0.03)	0.37	0.39	0.41	0.75 (± 0.03)	0.72	0.75	0.77
MetaLR	0.81 (± 0.01)	0.80	0.81	0.83	0.95 (± 0.01)	0.94	0.95	0.95
MutationAssessor	0.40 (± 0.04)	0.37	0.40	0.44	0.77 (± 0.03)	0.75	0.78	0.80
Reduced/parsimonious model								
Cluster weights (ω_k_):	0.35 (± 0.04)	0.28	0.35	0.43	0.65 (± 0.04)	0.57	0.65	0.72
Cluster means (µ_k_):								
CADD	13.2 (± 0.89)	11.5	13.2	14.9	24.2 (± 0.26)	23.7	24.2	24.6
REVEL score	0.35 (± 0.01)	0.33	0.35	0.37	0.73 (± 0.02)	0.69	0.73	0.77
MutationAssessor	0.36 (± 0.03)	0.30	0.36	0.41	0.76 (± 0.02)	0.72	0.76	0.78

**Table 3:** Full model confusion matrices and performance measures in the validation and test sets (DIC: 66897.27).

	Validation set (n = 99)		Test set (n = 98)	
	Reference		Reference	
	Pathogenic	Benign	Pathogenic	Benign
Predicted class				
Pathogenic	45	5	42	4
Benign	6	43	3	49
Metric				
Accuracy (95% CI)	0.89 (0.81–0.94)		0.93 (0.86–0.97)	
Balanced accuracy	0.89		0.93	
No-information rate	0.52		0.94	
P_acc > NIR_	< 0.0001		< 0.0001	
Sensitivity	0.88		0.93	
Specificity	0.89		0.92	
PPV	0.90		0.91	
NPV	0.88		0.94	
Cohen’s Kappa	0.78		0.86	
F_1_ score	0.89		0.92	

DIC, deviance information criterion; NPV, negative predictive value; PPV, positive predicted value.

Deviance information criterion (DIC)-based backward elimination reduced the model dimensionality to a three-parameter mixture model containing only the scores CADD, REVEL, and MutationAssessor. The reduced model had a similar performance and even outperformed the full model for some matrices (Table 4). The models' centroid estimates, predicted labels, and performance metrics were invariant to the seed and priors used.

**Table 4:** Parsimonious model confusion matrices and performance measures in the validation and test sets (DIC: 13545.79).

	Validation set (n = 99)		Test set (n = 98)	
	Reference		Reference	
	Pathogenic	Benign	Pathogenic	Benign
Predicted class				
Pathogenic	46	7	46	8
Benign	3	43	1	43
Metric				
Accuracy (95% CI)	0.90 (0.82–0.95)		0.91 (0.83–0.96)	
Balanced accuracy	0.90		0.91	
No-information rate	0.51		0.52	
P_acc > NIR_	< .0001		< .0001	
Sensitivity	0.94		0.98	
Specificity	0.86		0.84	
PPV	0.87		0.85	
NPV	0.94		0.98	
Cohen’s Kappa	0.80		0.82	
F_1_ score	0.91		0.91	

Upon cluster centroid of SIFT, PolyPhen-2, CADD, REVEL, MetaLR, and MutationAssessor of the full model (Table 2), we developed an Excel-based pathogenicity prediction tool that we called “SanfilippoPred”. “SanfilippoPred” considers 2.50%, 50.0%, and 97.5% percentiles for SIFT, PolyPhen, CADD, REVEL, MetaLR, and MutationAssessor. Supplementary (1) shows a brief user guide to how SanfilippoPred works and its requirements.

### SanfilippoPred outperforms existing genome-wide tools for the classification of known labeling NAGLU, GNS, HGSNAT and SGSH variants

**Figure 2:**
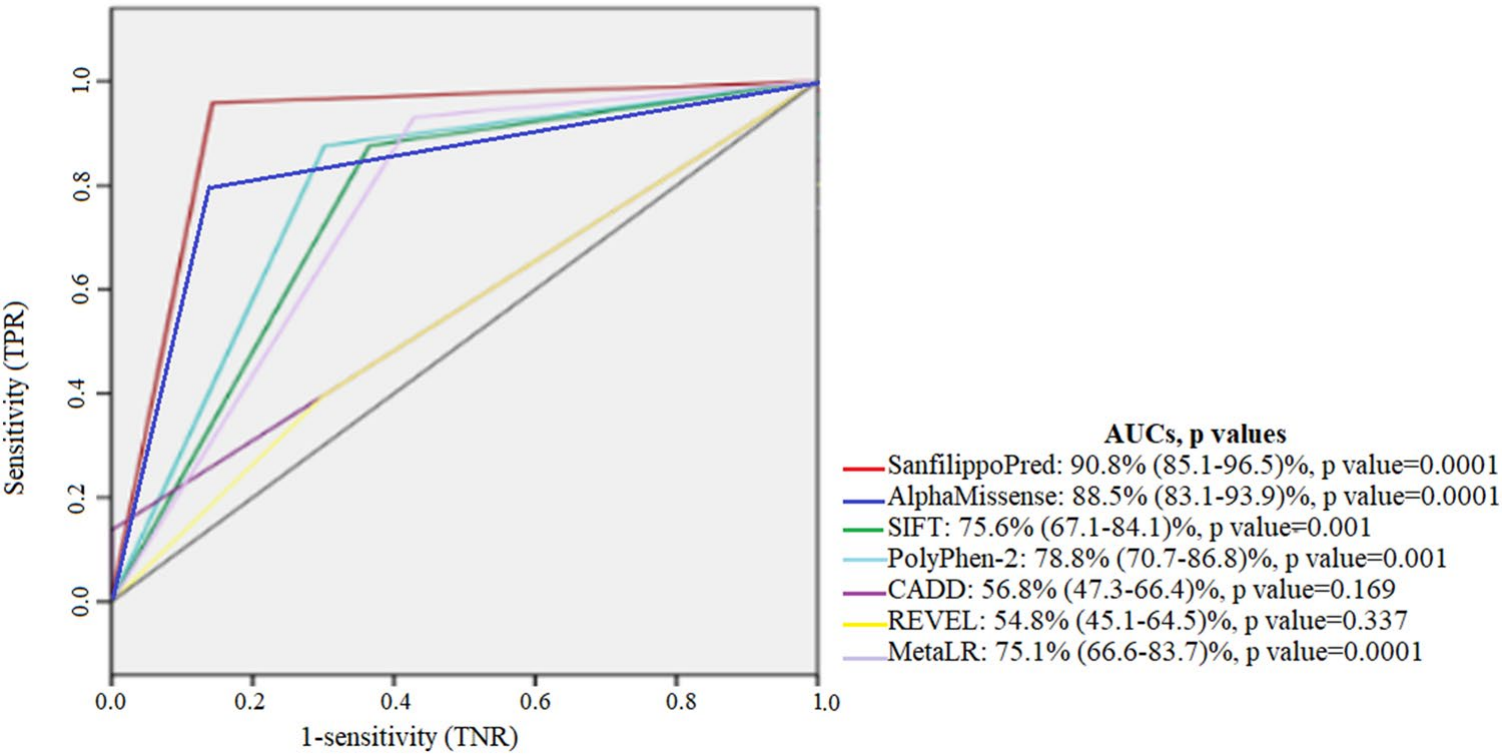
Comparison of receiver operating characteristic (ROC) curves of existing prediction scores and SanfilippoPred scores using all labelled missense variants of NAGLU, GNS, HGSNAT, and SGSH genes (n = 331). The AUCs showed that SanfilippoPred maximizes the identification of both pathogenic and benign variants in those genes.

Using the labelled variants of *NAGLU*, *GNS, HGSNAT,* and *SGSH* variants (n = 331), “SanfilippoPred” performance was compared to the individual genome-wide variant pathogenicity predictors REVEL, CADD, SIFT, Polyphen-2, MutationAssessor, and AlphMissense. “SanfilippoPred” demonstrated superior performance in pathogenicity prediction with an AUC of 0.908 (Fig. 2) and a pathogenicity probability threshold of ≥ 0.50.

### The clinical decision specificity of “SanfilippoPred” showed relative specificity towards known labelling MPS III-relevant variants

**Figure 3:**
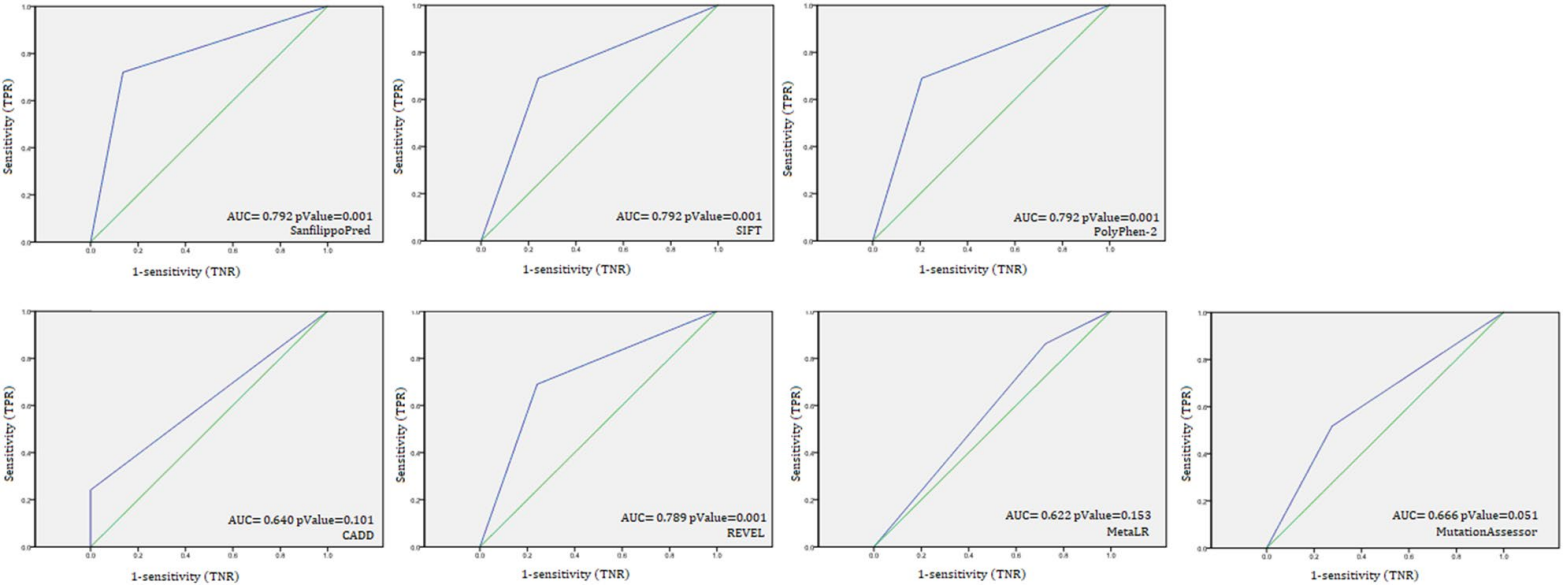
Receiver operating characteristic (ROC) curves of existing prediction scores and SanfilippoPred scores using all labelled missense variants of the Nkx2-5, GATA4, and COX10 genes. The AUCs showed that SanfilippoPred has powerful discrimination towards Sanfilippo-relevant variants.

To test the clinical decision specificity of MPS III using “SanfilippoPred”, we collected three additional labelled missense variant sets as an independent specificity test datasets. This dataset included 96 known pathogenicity variants for the *Nkx2-5, GATA4,* and *Cox10* genes that were registered in the Ensembl database with the criteria provided. The overall matrices of sensitivity, specificity, accuracy, and precision showed the relative specificity of our tool to MPS III-relevant variants, as shown in Fig. 3 and Table 5.

**Table 5:** Performance of SanfilippoPred tool against number of the wide-genome prediction tools.

ROC matrices	SanfilippoPred	SIFT	PolyPhen-2	CADD	REVEL	MetaLR	variantAssessor
Sensitivity	0.720	0.690	0.690	0.241	0.690	0.862	0.517
Specificity	0.864	0.759	0.793	1	0.759	0.276	0.724
Accuracy	0.787	0.724	0.741	0.621	0.724	0.569	0.621
Precision	0.857	0.741	0.769	1	0.741	0.543	0.652

To identify the sensitivity threshold of the SanfilippoPred tool, we used high-confidence pathogenic and likely-pathogenic *NAGLU* variants from the ClinVar database. Pathogenicity prediction probabilities of “SanfilippoPred” were highly concordant with consensus ClinVar classifications demonstrating 0.857 sensitivity. Therefore, 0.857 is considered a sensitivity threshold of the SanfilippoPred calculator tool.

## Discussion

The prevalence of increasingly large numbers of genetic variations led to the necessity of predictor and prioritize variant tools, especially since the vast majority of these variants are defined as variants of uncertain significance (VUS)^14,15^. Accurate pathogenicity prediction of missense variants is essential in genetic studies, clinical diagnosis, healthcare support, and clinical decision-making. Most approaches to predicting the functional effects or pathogenicity of missense variations rely on either sequence or structural information. However, machine learning is a new model to predict the pathogenicity of missense variants in the human genome^16^.

Our “SanfilippoPred” groups together the four genes associated with MPS III. These variants are missense with criteria provided and extracted from Ensembl 108, and they are clinically asserted from ClinVar, LOVD, VARCARDS, and ACMG (Franklin, Geenox) databases to avoid biased annotation in the individual database.

Based on our hypothesis that the MPS III-relevant variant pathogenicity prediction tools may be improved by using gene and disease-specific datasets supplemented with complementary multi-scale computational tools, we used a Bayesian Gaussian mixture model to explore the hidden relationships among eight computational scores for VUS missense variants of the *NAGLU* gene, using the score values as features in the training dataset. We developed “SanfilippoPred” to avoid the pitfalls of relying on genome-wide variant classifiers in variant pathogenicity interpretation and to support the clinical decision of MPS III over the currently available genome-wide tools. Validation and test datasets showed high sensitivity and specificity. Moreover, to predict the pathogenicity of *NAGLU* VUS variants,  in addition, “SanfilippoPred” showed a powerful tool for true pathogenic *NAGLU* variants with 85.7% sensitivity.

Supporting our hypothesis regarding disease-specific predictors is Zhang et al.^11^ disease-specific variant classifier, “CardioBoost”, which has high global discrimination accuracy (precision recall area under the curve [AUC] = 0.91 for cardiomyopathies and 0.96 for arrhythmias^11^. Similarly, Hutter et al.^17^ developed “HePPy” (Haematological Predictor of Pathogenicity) with impressive discrimination performance for somatic disease-causing variants in a haematological setting^17^. Furthermore, regarding gene-specific predictors as  reported by Li et al.^14^, their predictor tool called “vERnet-B” accurately predicts BRCA1 pathogenicity recognition through its tertiary structure features derived from the “AlphaFold2” tool^14^.

It is well known that the advantages of disease-specific training datasets are; (1) decreasing the false prediction of benign variants as disease-causing, and (2) establishing the relationship between pathogenicity impact and gene nature. It is worth noting that every group of genes  has distinct molecular mechanisms and biological functions. Zhang et al.^11^ pointed that disease pathogenicity–specific tools are most powerful in the genotype–phenotype relationship, and overcome the false negative pathogenicity rate of genome-wide tools because they are trained on universal labels^11^.

**Figure 4:**
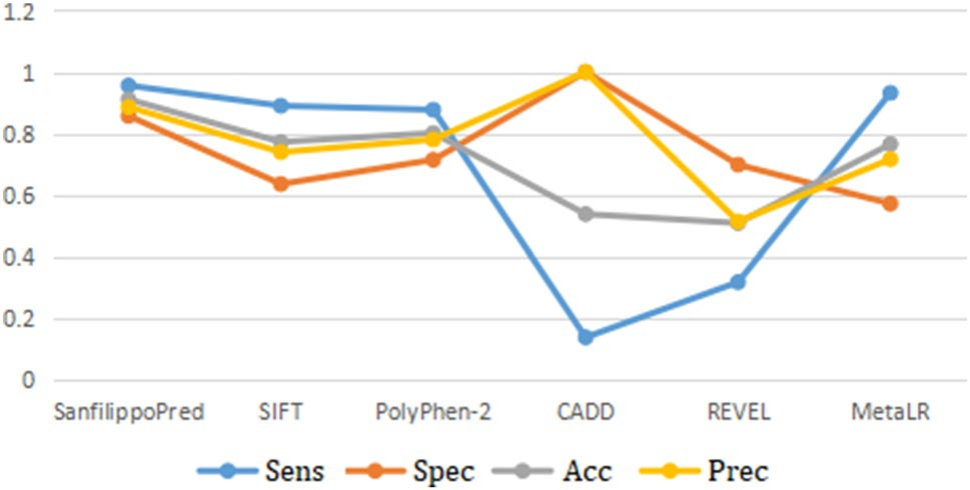
Receiver operating characteristic (ROC) curve matrices of our SanfilippoPred calculator tool and five individual predictors showing (1) consistent sensitivity, specificity, accuracy, and precision scores of the SanfilippoPred calculator tool, (2) relative performance convergence among SanfilippoPred and both SIFT and PolyPhen-2, and (3) outperforming the SanfilippoPred tool over five genome-wide tools.

Defect in one of the four genes; *NAGLU*, *GNS*, *HGSNAT*, and *SGSH* is causing four subtypes of MPS III. It is worth noting that we observed (1) consistent sensitivity, specificity, accuracy, and precision scores of SanfilippoPred, and (2) relative performance convergence between our tool and SIFT and PolyPhen-2 tools, as shown in Fig. 4. The consistent scores of the SanfilippoPred tool may reflect its interpretation stability and external validity. Also, the convergence manner between our SanfilippoPred,  SIFT, and PolyPhen-2 tools may refer to amino acid conservation and physiochemical proprieties distances, whose effect on the protein structure could play a significant role in the pathogenicity of MPS III variants. Interestingly, Clark et al.^18^ pointed out that sequence conservation is important to predict the impact of missense variants on the enzymatic activity of *NAGLU*^18^.

## Conclusions

Our study had some limitations that are; (1) we have considered the prediction of pathogenicity for only the missense variants, and other variant classes may consider low-confidence training data, (2) all included gene variants associated with MPS III subtypes only, (3) some pathogenicity evidence was neglected during SanfilippoPred modelling, such as minor allele frequency, (4) the SanfilippoPred calculator tool has constrain limits, such as indeterminate feature scores, and (5) we intended to adopt an unsupervised machine learning approach, so any different learning framework structure may give different output. “SanfilippoPred” is mainly developed to predict the pathogenicity of MPS III variants, not as a standalone clinical decision tool.

Finally, “SanfilippoPred” confirmed the hypothesis that a single pathogenic score cannot capture the true pathogenicity of a variant decision, and gene-disease-specific pathogenicity tools are recommended.

## Methods

### Missense variants extraction and training dataset

A total of 612 missense variants of the N-acetyl-alpha-glucosaminidase (*NAGLU*) gene were extracted from Ensembl 108 (accessed in  April 2021). All variants with controversial clinical significance and those associated with other diseases other than MPS III were excluded. All MPS III-relevant missense variants were asserted according to the status of reviewing as “criteria provided” from the submitter or “reviewed by expert panel” in the ClinVar, Ensembl, LOVD, VARCARDS (damage score equal to one for pathogenic, less than or equal to 0.3 for benign) and ACMG (Franklin, Genoox) databases. The asserted missense variants were categorized in two groups according to their clinical significance; (1) unlabelled variants that include 415 variants (67.8%) with VUS assertion, (2) labelled variants that include 101 variants (16.5%) with benign or likely benign assertion, and 96 variants (15.7%) with pathogenic or likely pathogenic assertion.

All 415 unlabelled missense variants formed the training dataset. All variants of the training dataset had known values of the pathogenicity prediction scores Polyphen-2, SIFT, REVEL, CADD, MetaLR, and MutationAssessor, in addition to Sneath's and Grantham's scores as conservation predictors. Parameters of extraction and clinical assertion are shown in Table 6.

**Table 6:** parameters of extraction and clinical assertion of *NAGLU* missense variants (n = 612).

Extraction resources	ensembl (Ensembl Release 108)
Clinical pathogenicity resources	ClinVar
	LOVD v. 3.0
	VARCARDS
	ACMG (Franklin, Genoox)
HGNC Symbol/ID	NAGLU/7632
Location	Ch17: 42,536,241-42,544,449
Transcript	ENST00000225927.7 NAGLU-201 (NM_000263.4)
Transcripts flag	MANE only
consequences	Missense
Clin. significance	VUS, benign, likely benign, pathogenic, likely pathogenic
Selected pathogenicity predictors	Polyphen-2, SIFT, REVEL, CADD, MetaLR, MutationAssessor, Sneath's and Grantham's scores

### Modeling

The analysis and modeling were performed using R software (version 4.1.1). Missing values in the training, validation and test datasets were imputed using the “MICE” package (version 3.13.0). The imputation method used was predictive mean matching (PMM).

Concerning the unlabeled variant data, a Bayesian Gaussian Mixture Model (BGMM) was fitted to cluster the data and obtain parameter estimates for each cluster with 95% credible intervals. The model was set to predict the probability of whether the variant belongs to one of the two clusters, whether the variant of interest is likely benign, or truly benign or likely pathogenic or truly pathogenic.

### Priors

A Gibbs sampler was manually coded to sample from the posterior distribution using conjugate priors on cluster weights, mean vectors, and covariance matrices; the prior inferred on cluster weights was a Dirichlet prior in the form ω_k_ ~ Dir (1, 1) for k ∈ {1, 2}, a multivariate normal prior on cluster means in the form of µ_k_ ~ *Ɲ*(ƞ, τ), where ƞ is the prior mean vector, which was taken as the mean vector of the observed data, and τ is the prior covariance and was taken as the covariance matrix of the observed data, scaled by a factor of 10, and an inverse Wishart prior on covariance matrices in the form of Ʃ_k_ ~ *W*_p_^−1^ (υ, Ψ), where υ is the prior degrees of freedom and Ψ is a *p* × *p* positive definite scale matrix, *p* is the number of parameters in the model, and the prior value of υ was taken as *p* and Ψ as the covariance matrix of the whole data.

To resolve the problem of label switching, a constraint was set on the cluster weights, such that ω_1_ < ω_2_, assuming that ω_1_ is the cluster weight for benign variants and ω_2_ for pathogenic variants (in other words, we assumed a priori that the probability that a variant is benign is less than the probability that it is pathogenic). Then, the MCMC output was reordered based on the weight constraint.

Backward elimination using the deviance information criterion (DIC) was performed to identify a parsimonious model. Both the full and parsimonious models were validated and tested on labelled data, and confusion matrix statistics (including accuracy, sensitivity, specificity, positive, and negative predictive values) were reported for each model. Validation and test data were partitioned equally, with an equal probability of sampling benign and pathogenic variants in each set.

Predictions were made based on the posterior predictive distribution using the following formula:

$$p_{{i,k,m}} = \frac{{\omega _{{k,m}} \left| {2\pi \left. {\sum\nolimits_{{k,m}} {} } \right|} \right.^{{ - \frac{1}{2}}} e^{{ - \frac{1}{2}}} \left( {x_{i} - \mu _{{k,m}} } \right)^{T} \sum\nolimits_{{k,m}}^{{ - 1}} {\left( {x_{i} - \mu _{{k,m}} } \right)} }}{{\sum\nolimits_{{k = 1}}^{K} {\omega _{{k,m}} \left| {2\pi \left. {\sum\nolimits_{{k,m}} {} } \right|} \right.^{{ - \frac{1}{2}}} e^{{ - \frac{1}{2}}} \left( {x_{i} - \mu _{{k,m}} } \right)^{T} \sum\nolimits_{{k,m}}^{{ - 1}} {\left( {x_{i} - \mu _{{k,m}} } \right)} } }}$$

where *p*_i, k, m_ is the probability that observation i belongs to cluster k for the Monte-Carlo sample m. For each m, the classification ĉ_i,m_ was obtained by argmax _k ∈ {1, 2}_ (*p*_i, k, m_), and the final classification ĉ_i_ was made by taking the mode of ĉ_i,m_.

### Model performance evaluation

The model was tested on the test and validation datasets, and the area under the curve (AUC), sensitivity, specificity, accuracy, precision, positive predictive value (PPV), negative predictive value (NPV), no-information rate (NIR), Cohen’s kappa, and the F1 score were calculated. Accuracy and NIR were compared using the exact binomial test (implemented by the caret package in R) calculated based on the results of receiver operating characteristic (ROC) curves. AUC measures the whole probability power of our ML predictor, sensitivity measures the true positive (pathogenic variants) rate by Sens. = TP/(TP + FN), specificity measures the true negative (benign variants) rate by Spec = TN/(TN + FP), accuracy measures the number of correct predictions, either positive or negative, by Acc. = (TN + TP)/(TN + TP + FN + FP), and precision measures rate of true positive belong to all positive predictions by Prec. = TP/(TP + FP).

To further evaluate our model, we extracted missense variants of the *GNS*, *HGSNAT* and *SGSH* genes using the same extraction parameters as the *NAGLU* gene variants. Then, we used our model to predict the pathogenicity of their variants, and the corresponding ROC curves were analysed.

### Genome-wide prediction tools for comparison

We compared the ROC curve data of our ML predictor for all extracted *NAGLU, GNS, HGSNAT,* and *SGSH* variants against individual REVEL, CADD, SIFT, Polyphen-2, MutationAssessor, and AlphMissense data for the same variants. Area under the curve (AUC), sensitivity, specificity, accuracy, and precision were measured.

96 known labelled variants for the *Nkx2-5, GATA4,* and *Cox10* genes (registered in the  Ensembl database with criteria) were extracted to test the clinical decision specificity of our ML model towards MPS III-relevant genes against non-MPS III-relevant ones.

### Evaluation of “SanfilippoPred” sensitivity

In June 2021, we accessed the ClinVar dataset and extracted pathogenic and likely pathogenic *NAGLU* variants with a two-star review status (i.e., criteria provided, multiple submitters, and no conflicts found). Twenty-one missense variants were collected to test the sensitivity threshold of “SanfilippoPred”.

### Supplementary Information

Supplementary Information.

## Data Availability

The datasets used and/or analysed during the current study are available from the corresponding author upon reasonable request.
