# Supplementary material for: Novel gene-specific Bayesian Gaussian mixture model to predict the missense variants pathogenicity of Sanfilippo syndrome
Source: Sci Rep. 2024 May 27;14:12148. doi: 10.1038/s41598-024-62352-0 (PMC11130188; doi:10.1038/s41598-024-62352-0)
Supplement: Supplementary file 1 — Supplementary Information. [file 41598_2024_62352_MOESM1_ESM.doc]

SanfilippoPred Software User Guide

(draft version)

1. **Introduction**

SanfilippoPred is friendly Excel-based software. It helps geneticists and clinicians to assess the binary pathogenicity spectrum (Benign vs Pathogenic) of *NAGLU, GNS, HGSNAT* and *SGSH* variants detected in clinically suspected Sanfilippo syndrome patients.

SanfilippoPred was derived from Machine Learning-based pathogenicity predictors model with accuracy of 0.93 (0.86-0.97 at CI 95%), sensitivity 0.93, and specificity 0.92.

SanfilippoPred workflow composed from;

- Open SanfilippoPred software
- Insert SIFT, PolyPhen-2, CADD, REVEL, MetaLR, MutationAssessor scores manually.
- Then, SanfilippoPred assessments will be calculated automatically.
- In graph section, the corresponding graph will be generated according to the scores of PROB-1, PROB-2, and PROB-T.
- PROB-T refers to the pathogenicity average with threshold ≥0.50. Pathogenicity Average (PA) Classify the query variants into benign or pathogenic annotation (see Table 1).

Table (1); Query variant annotation according to PA values.

| **Parameter** | **In the benign interface** | **In the pathogenic interface** | **Annotation** | **Color** |
| --- | --- | --- | --- | --- |
| **Pathogenicity average (PA)** | ≥ 0.5 | ≤0.5 | Predicted benign | Green shadow |
|  | ≤0.5 | ≥ 0.5 | Predicted pathogenic | Red shadow |
|  | >0.5 | >0.5 | Predicted VUS | colorless |
|  | =0.5 | =0.5 | Ambiguous | Green and Red |

**Note;** No installation required. SanfilippoPred will be available for free on the website. It will only require free registration.

1. **Requirements of Predictor Scores Input.**

To calculate SIFT, PolyPhen-2, CADD, REVEL, MetaLR, MutationAssessor scores of the query variant, the user should be access the following free web resources;

**SIFT:** https://sift.bii.a-star.edu.sg/

**PolyPhen-2:** https://genetics.bwh.harvard.edu/pph2/

**CADD:** https://cadd.gs.washington.edu/

**REVEL:** https://sites.google.com/site/revelgenomics/

**MetaLR:** https://sites.google.com/site/revelgenomics/

**MutationAssessor:** https://mutationassessor.org/r3/

**OR,** the user can be perform that via Variant Effect Predictor (VEP) immediately.

1. **SanfilippoPred Software Interface.**


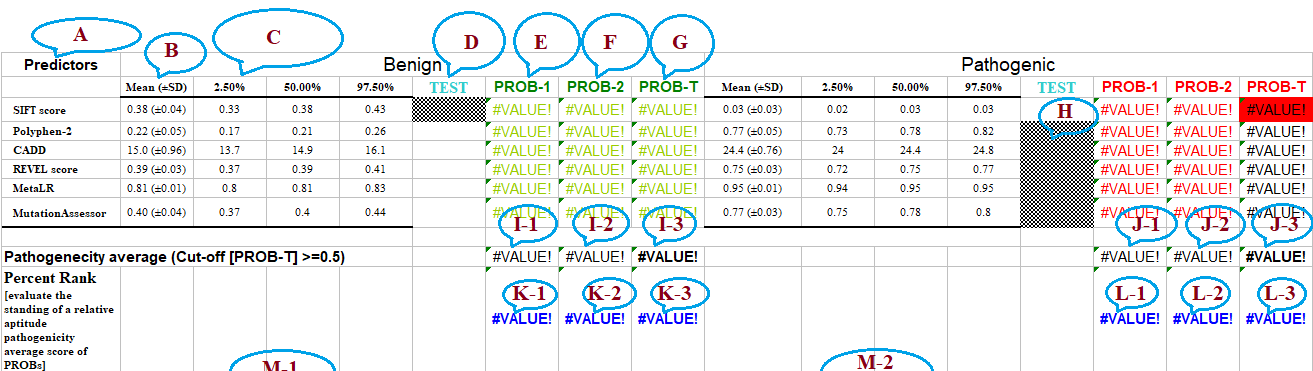


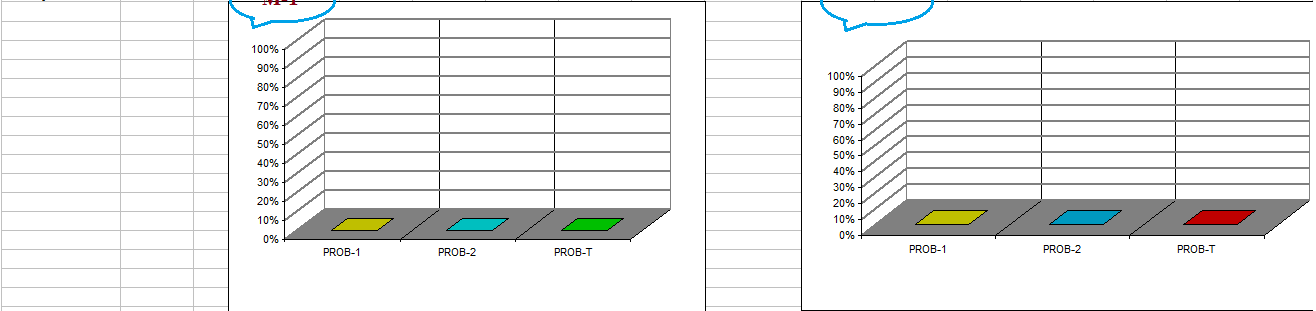


Fig.1: SanfilippoPred software interface.

Interface of the SanfilippoPred software consist of;

| **Symbol** | **Definition** |
| --- | --- |
| - Numerical section | |
| A | Pathogenicity predictors |
| B | Mean and standard deviation for each predictor |
| C | 2.50%, 50.0%, and 97.5% percentiles for each predictor |
| D | Scores of the query variant |
| E | Probability (PROB-1) expresses the fall of the score of the query variant (in D) within percentile of each predictor (in C). The committed values are scored by "1" and uncommitted values are scored by "0". |
| F | Probability (PROB-2) expresses the fall of the score of the query variant (in D) within the known severity scores of each predictor. The committed values are scored by "1" and uncommitted values are scored by "0". |
| G | PROB-1 + PROB-2 |
| H | 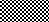 Shadow will be automatically showed above scores that out of PROB-1 and PROB-2 spectrum. It is referring to the neglected scores. |
| I-1/2/3 | Refers to average of calculated scores of PROB-1, PROB-2, and PROB-T respectively in benign estimation section.  I-3 score will be highlighted by green, if the PROB-T scores indicate to benign effect. |
| J-1/2/3 | Refers to average of calculated scores of PROB-1, PROB-2, and PROB-T respectively in pathogenic estimation section.  J-3 score will be highlighted by red, if the PROB-T scores indicate to pathogenic effect. |
| K-1/2/3 | Refers to relative contribution of PROB-1 and PROB-2 estimators in PROB-T inside benign estimation section. |
| L-1/2/3 | Refers to relative contribution of PROB-1 and PROB-2 estimators in PROB-T inside pathogenic estimation section. |
| - Graphical section | |
| M-1/2 | Converting the pathogenicity average values of PROB-1, PROB-2, and PROB-T into 3-D bars immediately. |

1. **Examples for Benign and pathogenic effect.**

**
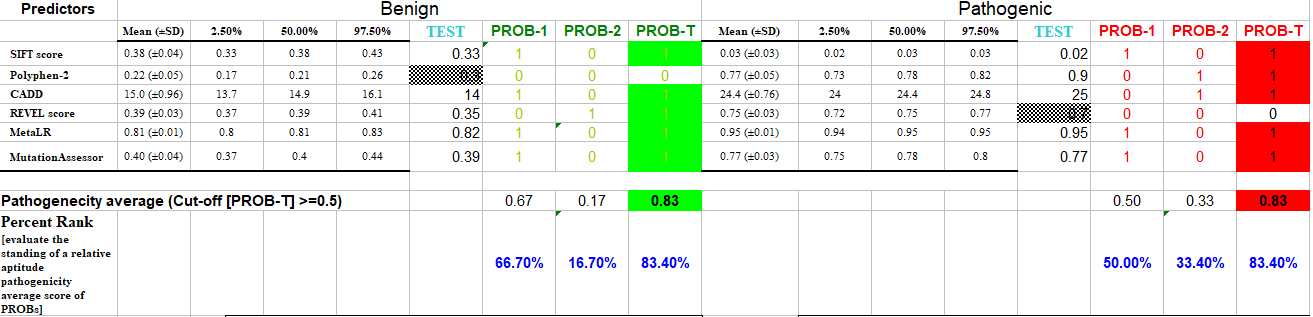

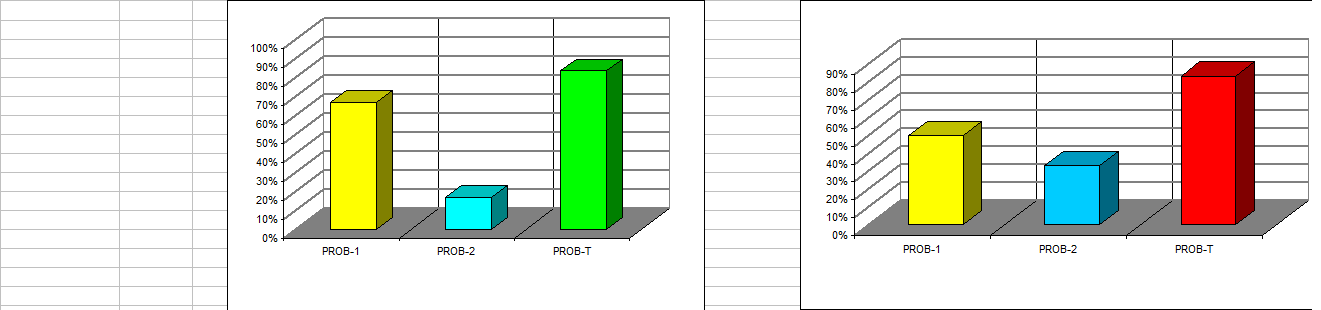
**
